# Complete Annotated Genome Sequences of Three *Campylobacter jejuni* Strains Isolated from Naturally Colonized Farm-Raised Chickens

**DOI:** 10.1128/genomeA.01407-16

**Published:** 2017-01-26

**Authors:** Michael E. Taveirne, Drew T. Dunham, Andrew Perault, Jessica M. Beauchamp, Steven Huynh, Craig T. Parker, Victor J. DiRita

**Affiliations:** a Department of Microbiology and Immunology, University of Michigan Medical School, Ann Arbor, Michigan, USA; b Department of Biological Sciences, North Carolina State University, Raleigh, North Carolina, USA; c Department of Microbiology and Molecular Genetics, Michigan State University, East Lansing, Michigan, USA; d Produce Safety and Microbiology Research Unit, Agricultural Research Service, U.S. Department of Agriculture, Albany, California, USA

## Abstract

*Campylobacter jejuni* is a leading cause of bacterially derived foodborne illness. Human illness is commonly associated with the handling and consumption of contaminated poultry products. Three *C. jejuni* strains were isolated from cecal contents of three different naturally colonized farm-raised chickens. The complete genomes of these three isolates are presented here.

## GENOME ANNOUNCEMENT

Campylobacters are a leading cause of human foodborne illness worldwide (1, 2). In the United States, *Campylobacter jejuni* is the predominant species associated with human illness, accounting for >99% of all reported infections (3 –5). Many wild, domesticated, and farm animals, especially birds, are carriers of *Campylobacter* species (6). Human *Campylobacter* infections are primarily due to the handling and consumption of contaminated poultry products, with water and milk also serving as sources of infection (7, 8). Molecular typing methods, including multilocus sequence typing and whole-genome sequencing, have been employed to better understand mechanisms of *Campylobacter* transmission from farm to fork (9–12).

To better understand the epidemiology of campylobacters in farm animals, specifically chickens, three *C. jejuni* isolates were isolated from three different Rainbow Ranger broiler chickens raised on a local farm in Dexter, MI. Cecal contents were plated on selective medium (Mueller-Hinton blood agar, supplemented with vancomycin [40 µg/ml], cefoperazone [40 µg/ml], trimethoprim [10 µg/ml], and cycloheximide [100 µg/ml]), and presumed *Campylobacter* isolates were confirmed by multiplex PCR (13).

Genome sequencing was performed using shotgun reads obtained on an Illumina MiSeq sequencer. Sequence reads with an average read length of 246 nucleotides (nt) were assembled *de novo* using the Roche Newbler assembler (version 2.3), resulting in 65 to 102 total contigs (>100 bp) per strain. A reference assembly against the *C. jejuni* MTVDSCj20 genome (accession no. CP008787) was performed within Geneious version 9.1. The *de novo* large contigs and the contigs derived from the reference assembly were used to create a draft scaffold. Scaffold gaps were filled using the small repeat *de novo* contigs and the Perl script Contig_extender3 (14). Homopolymeric GC tracts were characterized using the high-depth MiSeq reads.

Strains MTVDSCj07, MTVDSCj13, and MTVDSCj16 have circular genomes of 1,653 kb (232× coverage), 1,684 kb (219× coverage), and 1,785 kb (154× coverage), respectively. All three strains harbor at least one plasmid of 44.9 kb (pMTVDSCj07-1), 44.7 kb (pMTVDSCj13-1), 73.4 kb (pMTVDSCj13-2), and/or 42.7 kb (pMTVDSCj16-1). Protein-, rRNA-, and tRNA-encoding genes were identified as described previously (15). The genomes were annotated based on the genomes of the *C. jejuni* strains NCTC 11168, 81-176, and MTVDSCj20 (GenBank accession numbers AL111168.1, CP000538.1, and CP008787, respectively). Additional annotation was performed using Geneious, BLASTP comparisons to proteins in the NCBI nonredundant database, and the identification of Pfam domains (version 26.0 [16]).

The complete annotated genome sequences of MTVDSCj07, MTVDSCj13, and MTVDSCj16 encode 1,554, 1,615, and 1,729 chromosomal open reading frames, respectively. The strains harbor megaplasmids (pMTVDSCj07-1, pMTVDSCj13-1, and pMTVDSCj16-1) containing *tet*(O), which is associated with tetracycline resistance. MTVDSCj13 and MTVDSCj16 possess a type VI secretion system locus (17); in MTVDSCj13, these genes are harbored on a plasmid (pMTVDSCj13-2), and in MTVDSCj16, they are within a plasmid-like insertion island linked to an arginyl-tRNA. The three strains contain different lipooligosaccharide (LOS) biosynthetic regions that allow each to synthesize sialylated LOS (18) and distinct capsular polysaccharide biosynthesis loci.

### Accession number(s).

The GenBank accession numbers for the *Campylobacter jejuni* strains are CP017031 and CP017416 for MTVDSCj07; CP017032, CP017418, and CP017417 for MTVDSCj13; and CP017033 and CP017419 for MTVDSCj16.
